# LPS-preconditioned mesenchymal stromal cells modify macrophage polarization for resolution of chronic inflammation via exosome-shuttled let-7b

**DOI:** 10.1186/s12967-015-0642-6

**Published:** 2015-09-19

**Authors:** Dongdong Ti, Haojie Hao, Chuan Tong, Jiejie Liu, Liang Dong, Jingxi Zheng, Yali Zhao, Huiling Liu, Xiaobing Fu, Weidong Han

**Affiliations:** Institute of Basic Medicine Science, College of Life Science, Chinese PLA General Hospital, No. 28 Fuxing Road, Beijing, 100853 China; Central Laboratory, Hainan Branch of Chinese PLA General Hospital, Sanya, 572013 China

**Keywords:** Mesenchymal stromal cells, Macrophage polarization, Exosome, LPS preconditioning

## Abstract

**Background:**

Within the last few years, it has become evident that LPS-preconditioned mesenchymal stromal cells (LPS pre-MSCs) show enhanced paracrine effects, including increased trophic support and improved regenerative and repair properties. MSCs may release large amounts of exosomes for cell-to-cell communication and maintain a dynamic and homeostatic microenvironment for tissue repair. The present study assesses the therapeutic efficacy and mechanisms of LPS-preconditioned MSC-derived exosomes (LPS pre-Exo) for chronic inflammation and wound healing.

**Methods:**

We extracted exosomes from the supernatant of LPS pre-MSCs using a gradient centrifugation method. In vitro, THP-1 cells were cultured with high glucose (HG, 30 mM) as an inflammatory model and treated with LPS pre-Exo for 48 h. The expression of inflammation-related cytokines was detected by real-time RT-PCR, and the distribution of macrophage subtype was measured by immunofluorescence. Next, the miRNA expression profiles of LPS pre-Exo were evaluated using miRNA microarray analysis. The molecular signaling pathway responsible for the regenerative potential was identified by western blotting. In vivo, we established a cutaneous wound model in streptozotocin-induced diabetic rats, and LPS pre-Exo were injected dispersively into the wound edge. The curative effects of LPS pre-Exo on inflammation and wound healing were observed and evaluated.

**Results:**

LPS pre-Exo have a better ability than untreated MSC-derived exosomes (un-Exo) to modulate the balance of macrophages due to their upregulation of the expression of anti-inflammatory cytokines and promotion of M2 macrophage activation. Microarray analysis of LPS pre-Exo identified the unique expression of let-7b compared with un-Exo, and the let-7b/TLR4 pathway served as potential contributor to macrophage polarization and inflammatory ablation. Further investigation of the mechanisms that control let-7b expression demonstrated that a TLR4/NF-κB/STAT3/AKT regulatory signaling pathway plays a critical role in the regulation of macrophage plasticity. Knockdown of AKT in THP-1 cells similarly abolished the immunomodulatory effect of LPS pre-Exo. In vivo, LPS pre-Exo greatly alleviated inflammation and enhanced diabetic cutaneous wound healing.

**Conclusion:**

LPS pre-Exo may have improved regulatory abilities for macrophage polarization and resolution of chronic inflammation by shuttling let-7b, and these exosomes carry much immunotherapeutic potential for wound healing.

**Electronic supplementary material:**

The online version of this article (doi:10.1186/s12967-015-0642-6) contains supplementary material, which is available to authorized users.

## Background

Impairment of cutaneous wound healing represents a serious complication of diabetes and is associated with significant morbidity and a massive socioeconomic burden [1]. Evidence suggests that chronic cutaneous wounds are characterized by an abnormal inflammatory state, with prolonged accumulation of macrophages and an increased release of pro-inflammatory cytokines [2, 3]. Therefore, orchestrating the inflammatory response might be a promising strategy to promote proper wound healing.

As the primary effectors of inflammation in tissue injury, macrophages can be polarized into the “classically activated” M1 phenotype or the “alternatively activated” M2 phenotype according to signals in the microenvironment [4, 5]. The proinflammatory responses of M1 macrophages depend on Toll-Like Receptors (TLRs) and the activation of NFκB, leading to pathogen phagocytosis, oxidative burst and intracellular killing. Conversely, activation of M2 macrophages leads to the recruitment of STAT3 or other transcription factors, resulting in dampened inflammation and promoting tissue remodeling. Previous studies have shown that in diabetic mouse models, high glucose can enhance unrestrained M1 cells but impair M2 cell polarization, produce a large number of pro-inflammatory mediators, and drive chronic inflammation [6]. Furthermore, dysregulated inflammation in diabetic mellitus allows for the hyper-induction of M1 macrophages and the aberrant production of inducible nitric oxide synthase (iNOS), causing de novo tissue destruction and recapitulating chronic wounds [7]. Therefore, the appropriate balancing of macrophage polarization plays a crucial role in regulating inflammation and subsequently accelerates tissue repair and homeostasis.

Regenerative medicine strategies using mesenchymal stromal cells (MSCs) are expected to be a hopeful alternative approach to the treatment of a wide variety of pathological conditions, such as chronic inflammation. Pelizzo and colleagues found that MSCs may relieve the inflammatory response by inducing the wound’s capacity to progress in regeneration of skin architecture and not regress to a chronic wound state, and this biological property of MSCs to promote the transition from inflammatory to the proliferative phase is very crucial for treating wounds where high levels of inflammation inhibit healing [8]. Furthermore, MSCs switched from infiltration of pro-inflammatory to anti-inflammatory macrophages for alleviating inflammation and augmenting cardiac regeneration [9]. In a sepsis model, MSCs can secrete certain growth factors to increase the percentage of reparative M2 macrophages and improve organ function [10]. Studies have found that the exposure of MSCs to the pharmacological agent lipopolysaccharide (LPS) can increase their trophic effects and functional properties to defend against the harsh inflammatory environment [11–13]. Liu et al. have confirmed that LPS-primed MSCs have a superior therapeutic ability to preserve skin flap survival in a diabetic rat model compared to unprimed MSCs [14]. However, it remains unclear how LPS-preconditioned MSCs (LPS pre-MSCs) resolve chronic inflammation and whether they may function by accommodating macrophage polarization.

Increasing data indicate that MSCs may create an optimal microenvironment for reducing inflammation and promote tissue repair through a paracrine mechanism, and exosomes play an important role in this process [15–17]. Exosomes are small membranous vesicles that contain bioactive molecules, including protein, messenger RNAs (mRNAs) and microRNAs (miRNAs), which can be transferred between cells and thus modulate cellular activities and reprogram the phenotype in recipient cells [18, 19]. As master molecular switches, miRNAs exchange bio-information between neighboring cells and mediate exosomal intercellular communication. Lin and colleagues confirmed that MSCs transferred exosomal miR-124 to astrocytes, enhanced their anti-inflammatory effects, and benefited neurite remodeling and functional recovery by increasing the expression of glutamate transporters [20]. Moreover, the induction of miR-146a may be protective against lung injury by suppressing TLR4-induced NF-κB-regulated gene expression and promoting the M2 macrophage phenotype in acute respiratory distress syndrome [21]. However, the roles of miRNA in exosome-derived LPS pre-MSCs (LPS pre-Exo) for macrophage polarization and suppression of chronic inflammation have not been elucidated.

In the present study, we successfully isolated and identified LPS pre-Exo. Using microarray analysis, we found that let-7b in LPS pre-Exo has dramatic effects on the regulation of macrophage plasticity to resolve chronic inflammation and enhance cutaneous wound healing. It is feasible that innovative ‘exosome-based therapy’ may be developed to reduce the difficulties and risks associated with whole MSC transplantation for the promotion of wound healing.

## Methods

This study was approved by the human ethics committees of the Chinese PLA General Hospital, Beijing, People’s Republic of China, and written informed consent was obtained from all subjects before umbilical cord collection.

### Isolation, culture and LPS-preconditioning of MSCs

Human umbilical cord MSCs (UC-MSCs) were isolated in our laboratory by processing human umbilical cord tissue as described by Professor Hou et al. [22]. Then, the cells were routinely resuspended in low-glucose Dulbecco’s modified Eagle’s medium (DMEM; Gibco Life Technologies, USA) supplemented with 10 % fetal bovine serum (Hyclone Laboratories, USA) and 100 U/ml penicillin/streptomycin (Gibco Life Technologies, USA). The cultures were maintained at 37 °C in 5 % CO_2_ and 95 % humidity, and cells at the 4th–5th passages were used for the subsequent experiments.

For LPS preconditioning, 1.5 × 10^6^ UC-MSCs per 15-cm cell culture dish were seeded for 24 h to achieve a confluence of 70–80 %. After the medium was aspirated, the cells were rinsed three times with PBS and treated with LPS (100 ng/ml in serum-free medium, Sigma, USA) or serum-free medium alone as a negative control and then incubated for 2 days prior to supernatant collection.

### Biological properties of LPS pre-MSCs

The morphology of LPS pre-MSCs was observed under a phase-contrast microscope (Olympus IX71). Their immunophenotype was determined by flow cytometry evaluating CD11a, CD34, CD73, CD90, CD105, and HLA-DR, and their multipotency was confirmed by osteogenic and adipogenic differentiation.

Cell viability after LPS treatment was evaluated using an Apoptosis Kit following the manufacturer’s instructions (BD Biosciences). Briefly, the cells were dyed with Annexin V and 7-AAD and analyzed using flow cytometry.

### Exosome extraction and identification

Exosomes were harvested from the supernatants of LPS pre-MSCs according to the protocol previously described [23, 24]. The cell supernatants were filtered through a 0.22-µm filter to remove large debris and dead cells, centrifuged at 10,000×*g* for 30 min to remove cellular debris, and then centrifuged at 100,000×*g* for 3 h at 4 °C. Following this step, the pellets primarily contained exosomes. The exosome pellets were resuspended in the appropriate buffer for protein or RNA analysis.

The exosomes were identified by the marker proteins CD9, CD63 or CD81 using western blotting [25], as well as by using a transmission electron microscope (TEM, Hitachi H-7650) to verify the exosome presence. The protein concentration of the exosomal fraction was quantified with the BCA protein assay kit following the manufacturer’s instructions (Pierce, USA).

### THP-1 cell culture and treatment

The human monocytic cell line THP-1 was purchased from the American Type Culture Collection (ATCC, Manassas, VA, USA). THP-1 cells were cultured in RPMI 1640 medium (Gibco Life Technologies, CA, USA) supplemented with 10 % FBS. The cells were grown at a density of 3 × 10^5^–6 × 10^5^ cells/ml as recommended by the ATCC. Then, the THP-1 cells were cultured with two concentrations (5 and 30 mM) of glucose in six-well plates, and differentiation was induced by treatment with phorbol 12-myristate 13-acetate (PMA, 160 ng/ml, Sigma). After 3 days, the non-adherent cells were removed with three rinses with PBS. Adherent cells were further incubated with fresh medium containing untreated MSC-derived exosomes (un-Exo, 20 μg/ml) or LPS pre-Exo (20 μg/ml) for an additional 48 h.

### Internalization of LPS pre-Exo into THP-1 cells

To trace LPS pre-Exo by fluorescent microscopy, they were labeled with DiI dye (Sigma) and washed in PBS with centrifugation at 100,000×*g* for 1 h at 4 °C. Then, the DiI-labeled LPS pre-Exo were co-cultured with THP-1 cells at a final concentration of 10 μg/ml. After 6 h, the cells were stained with Hoechst33342 for 8 min and washed with PBS. Finally, the cells were examined and photographed with a confocal imaging system (Olympus FV1200).

### Quantitative real-time polymerase chain reaction (RT-PCR)

Total mRNA was isolated from treated THP-1 cells using Trizol reagent (Takara) and reverse transcribed into cDNA using a cDNA Synthesis Kit (Takara) according to the manufacturer’s protocol. Then, RT-PCR was performed using targeted gene primers (Invitrogen, San Diego, CA, USA) following the manufacturer’s cycling parameters and run on an ABI Prism 7500 Sequence Detection System (Applied Biosystems) using SYBR Green Mastermix (Toyobo). The primer sequences are shown in Additional file 1: Table S1. Relative fold changes in expression were calculated by normalizing to a housekeeping gene (GADPH) to adjust for loading variation.

### Exosomal miRNA isolation, microarray and quantification

RNA was extracted from different exosomes using the Total Exosome RNA and Protein Isolation Kit (Invitrogen, USA) and mirVana RNA Isolation Kit (Ambion, USA) according to the manufacturer’s protocols. Total RNA was quantified by the NanoDrop ND-2000 (Thermo Scientific) and the RNA integrity was assessed using Agilent Bioanalyzer 2100 (Agilent Technologies).

For microarray analysis, 100 ng of total RNA was labeled with pCp-Cy3 using the Agilent miRNA labeling reagent and then hybridized with Agilent Human miRNA Microarray (Release 19.0, Agilent, CA, USA), which contains 60,000 probes for 1888 human microRNAs (miRBase 19.0). The hybridization signals were detected with the Agilent G2505C Microarray Scanner System, and the scanned images were analyzed using Feature Extraction Software (version10.7.1.1, Agilent Technologies). Data analysis was performed using GeneSpring GX 12.5 software (Agilent Technologies). The raw data was normalized with the quantile algorithm. The probes that at least one condition out of two conditions have flags in “Detected” were chosen for further data analysis. Differentially expressed miRNAs were then identified through fold change as well as p value calculated by t test, including correction for multiple testing using the False Discovery Rate (FDR) method. The threshold set for up- and down-regulated genes was a fold change ≥2.0 and a p value ≤0.05. To confirm the results from microRNA profiling, candidate miRNAs were quantified by RT-PCR using TaqMan microRNA kits (Life Technologies). MiRNA relative expression was normalized against U6 snRNA as an endogenous control.

### MiRNA target prediction and pathway enrichment analysis

The target genes of miRNAs were predicted by the public web-based prediction tools TargetScan (http://www.targetscan.org/) and miRanda (http://www.microrna.org). The predicted genes of candidate miRNAs were identified using DAVID Bioinformatics Database functional-annotation tools (http://david.abcc.ncifcrf.gov/) for their functional annotation clustering analysis. The predominant biological pathways for the selected miRNAs were identified.

### Transfection of miR mimics and inhibitors

THP-1 cells were treated with LPS pre-Exo or un-Exo in 6-well culture plates and transfected with the indicated miRNA mimics, inhibitors or negative controls using Lipofectamine 2000 (Invitrogen) according to the manufacturer’s instructions. The cells were harvested after 48 h of transfection for subsequent experiments.

### Diabetic cutaneous wound preparation

All procedures were performed according to the Chinese PLA General Hospital Animal Care and Use Committee’s guidelines for the principles of animal care. Diabetic cutaneous wounds were prepared as described by Professor Hao et al. [26]. The animals were divided into the following four groups (n = 6): (1) the Normal group (Nor), (2) the Diabetic group (Dia), (3) the Diabetic + un-Exo group (Dia + un-Exo), and (4) the Diabetic + LPS pre-Exo group (Dia + LPS pre-Exo).

### Histology

Cutaneous wound bed tissues at 3 days were excised and fixed in 4 % phosphate-buffered formalin (pH 7.4), embedded in paraffin, sectioned at 5 µm, and mounted on adhesive glass slides. Then, the sections were stained with hematoxylin and eosin (H&E) using standard procedures and observed under a light microscope (Olympus BX53).

### Immunofluorescence

After fixation with paraformaldehyde and permeabilized with 0.1 % Triton X-100, THP-1 cells or cutaneous excised tissues were incubated with the desired primary antibodies against the M1 marker iNOS (Abcam) and the M2 marker Arg-1 (Abcam) overnight at 4 °C and with Alexa Fluor-conjugated IgG secondary antibodies (1:300; Santa) for 60 min at room temperature, followed by counter-staining of the nucleus with Hoechst33342 dye (Sigma) for 5 min. Finally, the cells were examined and photographed with a confocal imaging system (Olympus FV1200). For quantification, 10 different fields from each sample preparation were randomly selected, and then the iNOS-, Arg-1- and Hoechst-positive areas were generated using Image-Pro Plus software (Media Cybernetics). The presence of various macrophage subtypes was expressed as the ratio between iNOS-/Arg-1-positive areas and Hoechst-positive regions.

### Western blotting

Proteins were isolated from treated THP-1 cells or cutaneous excised tissues using radioimmunoprecipitation buffer with protease inhibitors (Sigma). Protein concentrations were determined by a Bradford assay (Pierce). The total protein (30 μg) of each sample was subjected to SDS-PAGE and immunoblotting with the desired antibodies against TLR4 (Abcam), STAT3 (Cell Signaling), p-STAT3 (Cell Signaling), AKT (Cell Signaling), p-AKT (Cell Signaling), NF-κB (Cell Signaling), p-P65 (Cell Signaling) and β-actin (Abcam), as previously described [27].

### Statistical analysis

All results are presented as the mean ± standard deviation from at least three independent experiments. Data analysis was performed using SPSS version 14.0.1 for Windows. Statistical significance was determined by Student’s t tests or one-way analysis of variance to evaluate the LPS pre-Exo treatment effect. Generally, group differences at the level of *p* < 0.05 were considered statistically significant.

## Results

### Characterization of LPS pre-MSCs

**Fig. 1:**
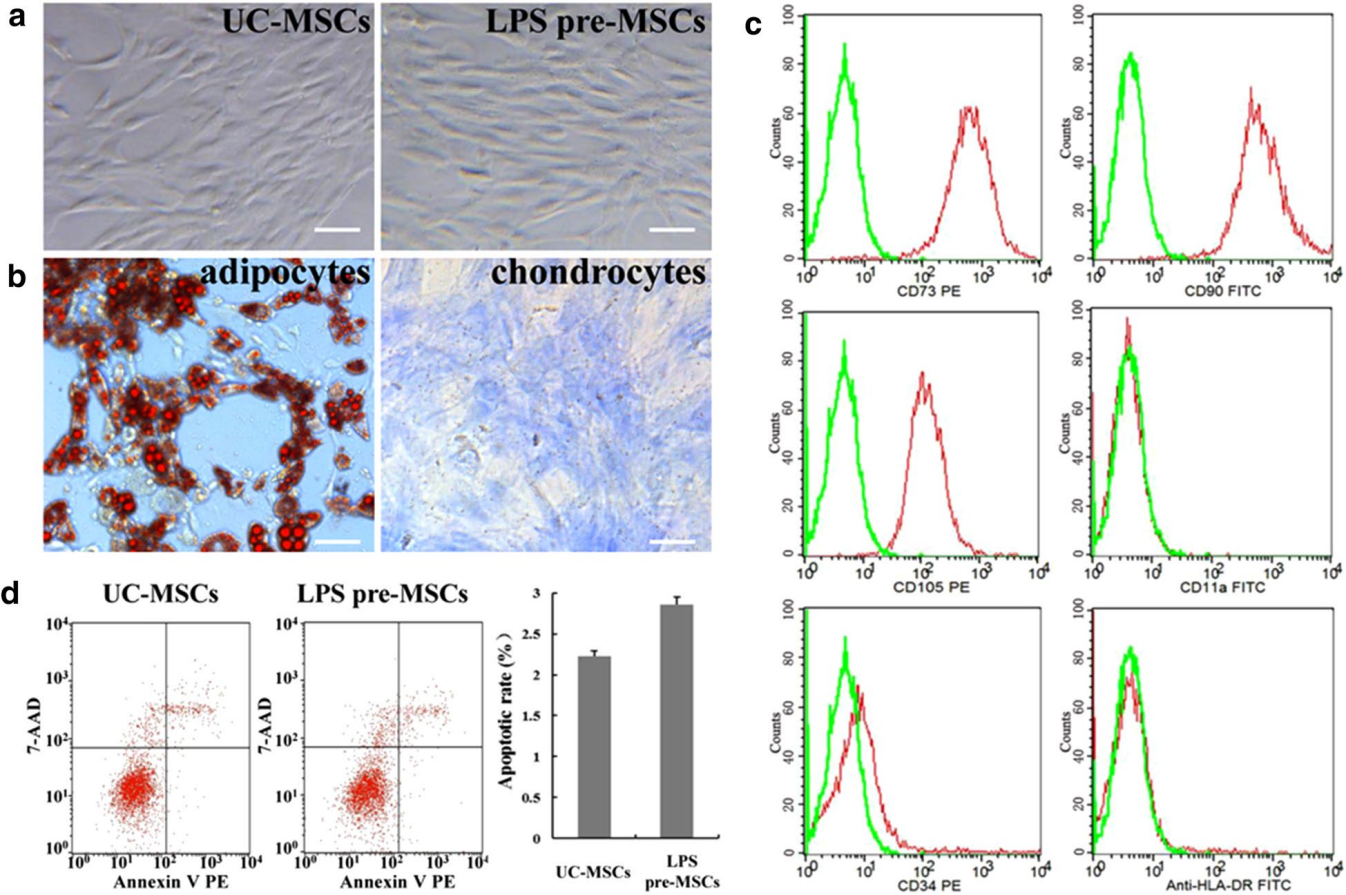
Characterations of LPS pre-MSCs. **a** Representative photomicrographs of UC-MSCs and LPS pre-MSCs in culture displaying similar spindle shaped morphology. *Scale bar* 100 μm. **b** LPS pre-MSCs differentiation into adipocytes and chondrocytes. **c** Immunophenotype of LPS pre-MSCs by flow cytometry. **d** After appropriate LPS stimulation, UC-MSCs apoptosis didn’t show clearly change by flow cytometry analysis of Annexin V and 7-AAD staining. Data are presented as the mean ± SEM of three separate experiments

Previous studies have shown that LPS preconditioning of MSCs enhanced the paracrine ability to improve the therapeutic potentiality [28]. We first evaluated the characteristics of LPS pre-MSCs. UC-MSCs were isolated from human umbilical cord tissue and pretreated with 100 ng/ml LPS for 2 days. After LPS treatment, UC-MSCs exhibited a spindle-shaped morphology and were positive for CD73, CD90 and CD105 and negative for CD11a, CD34, and HLA-DR by FACS analysis (Fig. 1a, c). Additionally, LPS pre-MSCs were found to be capable of differentiating into osteoblasts and adipocytes (Fig. 1b). Annexin V/7-AAD staining showed that no significant induction of apoptosis occurred in MSCs upon exposure to LPS (Fig. 1d). These data confirm that LPS pre-MSCs have a similar pattern regarding the extent and level of original MSC features.

### Validation of LPS pre-Exo

**Fig. 2:**
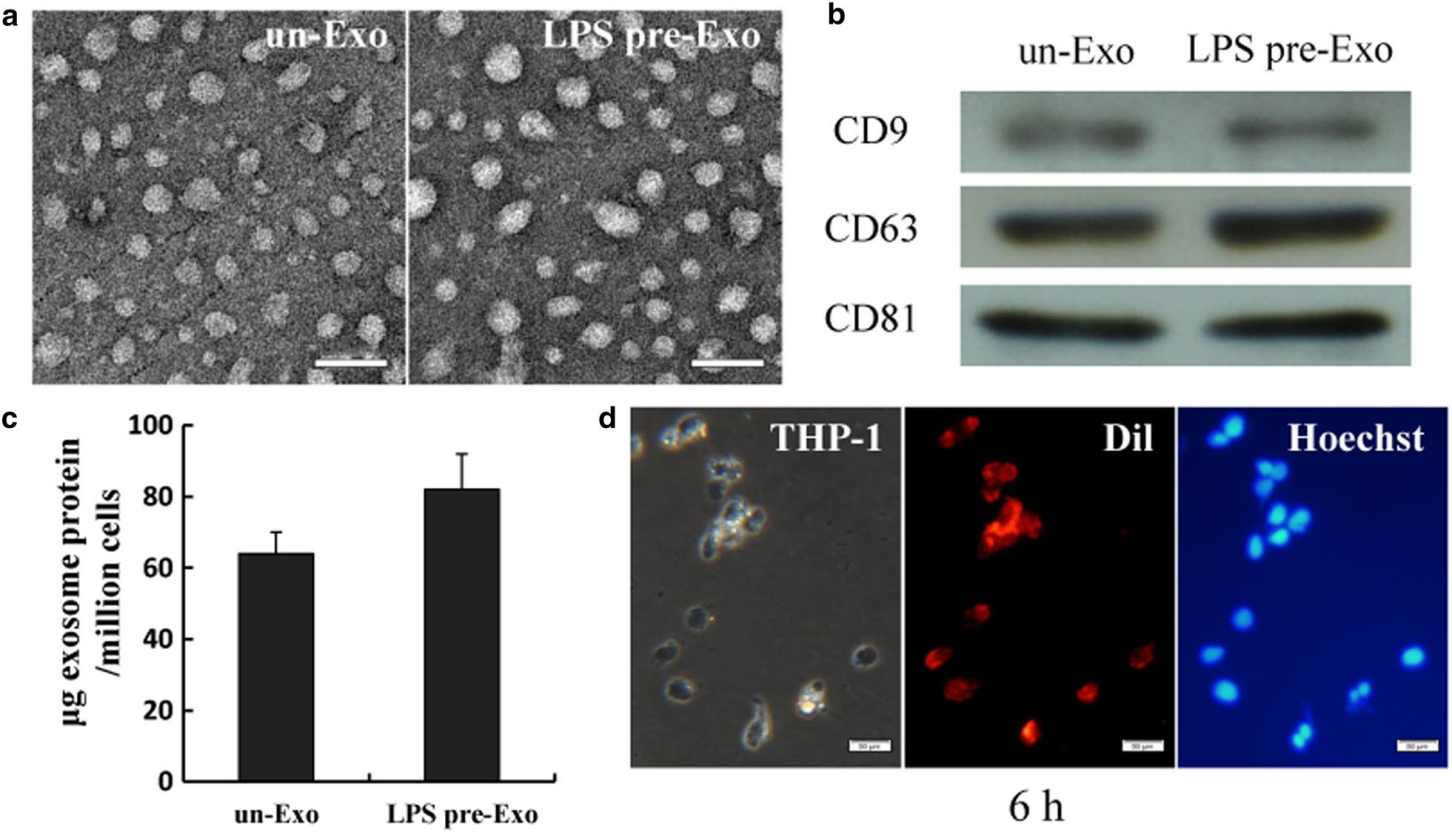
Identification of LPS pre-Exo. **a** Transmission electron micrograph of exosomes derived from diverse processing MSCs. *Scale bar* 100 nm. **b** Detection of CD9, CD63, and CD81 expression in MSC derived exosomes by western blotting. **c** UC-MSCs showed enhanced exosome secretion after LPS preconditioning. Data are presented as the mean ± SEM of three separate experiments. **d** Fluorescence microscopy detection of the uptake of Dil-labeled exosomes (*red*) by THP-1 cells (Hoechst *blue*) for 6 h. *Scale bars* 50 µm

Because exosomes derived from MSCs have a vital role in orchestrating wound healing, we extracted exosomes from the supernatants of LPS pre-MSCs using a gradient centrifugation method. LPS pre-Exo were identified as small vesicles ranging from 40 to 90 nm in a cup-shaped form and expressed exosomal markers such as CD9, CD63 and CD81 (Fig. 2a, b). Then to estimate levels of exosome release, the protein concentration of purified preparations were assessed by BCA methods and normalized to the number of manipulated cells. In our experiments, we routinely isolated approximately 410 µg protein equivalent of exosomes from 80 ml of the supernatants collected from cultures of 5 × 107 LPS pre-MSCs. And we found a significantly greater level (37 %) of total proteins in LPS pre-Exo than in un-Exo, implying that LPS stimulation increased the secretion of exosomes from UC-MSCs (Fig. 2c). Additionally, DiI-exosomes were internalized in THP-1 cells and localized in the cytoplasm, as visualized by confocal microscopy (Fig. 2d). These data showed that we successfully obtained LPS pre-Exo.

### LPS pre-Exo converted inflammatory THP-1 cells to M2 polarization

**Fig. 3:**
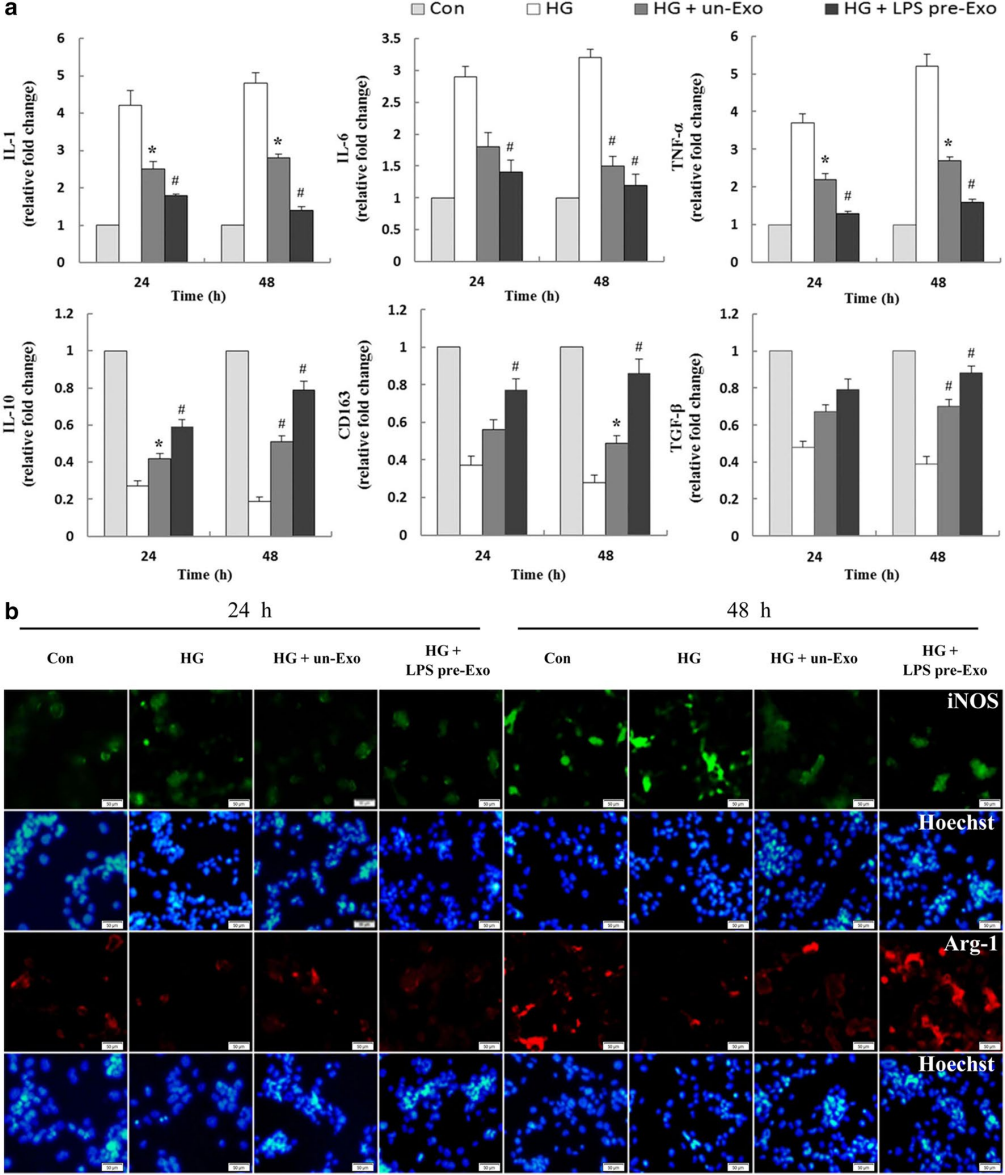
LPS pre-Exo converted inflammatory THP-1 cells to M2 polarization. **a** Pro-inflammatory cytokines (IL-1, IL-6, TNF-α), anti-inflammatory cytokines (IL-10, TGF-β) and M2 macrophages surface marker CD163 expression in THP-1 cells after culturing with LPS pre-Exo for various times. mRNA expression of interest genes is normalized to GADPH and given as relative change. Data are presented as the mean ± SEM of three separate experiments. *Compared with Con, *p* < 0.05;^**#**^compared with HG, *p* < 0.05. **b** Immunofluorescence analysis of macrophage phenotype, and iNOS (M1 *green*) or Arg1 (M2 *red*) were expressed in THP-1 cells (Hoechst *blue*). *Scale bar* 50 µm

Because high glucose induces macrophage into a pro-inflammatory state [29], we examined the influence of LPS pre-Exo on THP-1 cells in a high-glucose environment. The RT-PCR results showed that after LPS pre-Exo treatment, THP-1 cells dramatically produced more anti-inflammatory cytokines (IL-10, TGF-β) and M2 macrophages surface marker CD163, and less pro-inflammatory cytokines, including IL-1, IL-6, and TNFα at 48 h (*p* < 0.05) (Fig. 3a). Furthermore, immunofluorescence staining showed that the density and distribution of M2 macrophages was significantly increased and M1 macrophages were markedly reduced in the LPS pre-Exo group, especially at 48 h (Fig. 3b). Taken together, these data indicate that LPS pre-Exo are a homeostatic regulator of macrophage polarization and facilitate the differentiation of macrophages to M2, but not M1.

### Unique expression pattern of miRNA in LPS pre-Exo

**Fig. 4:**
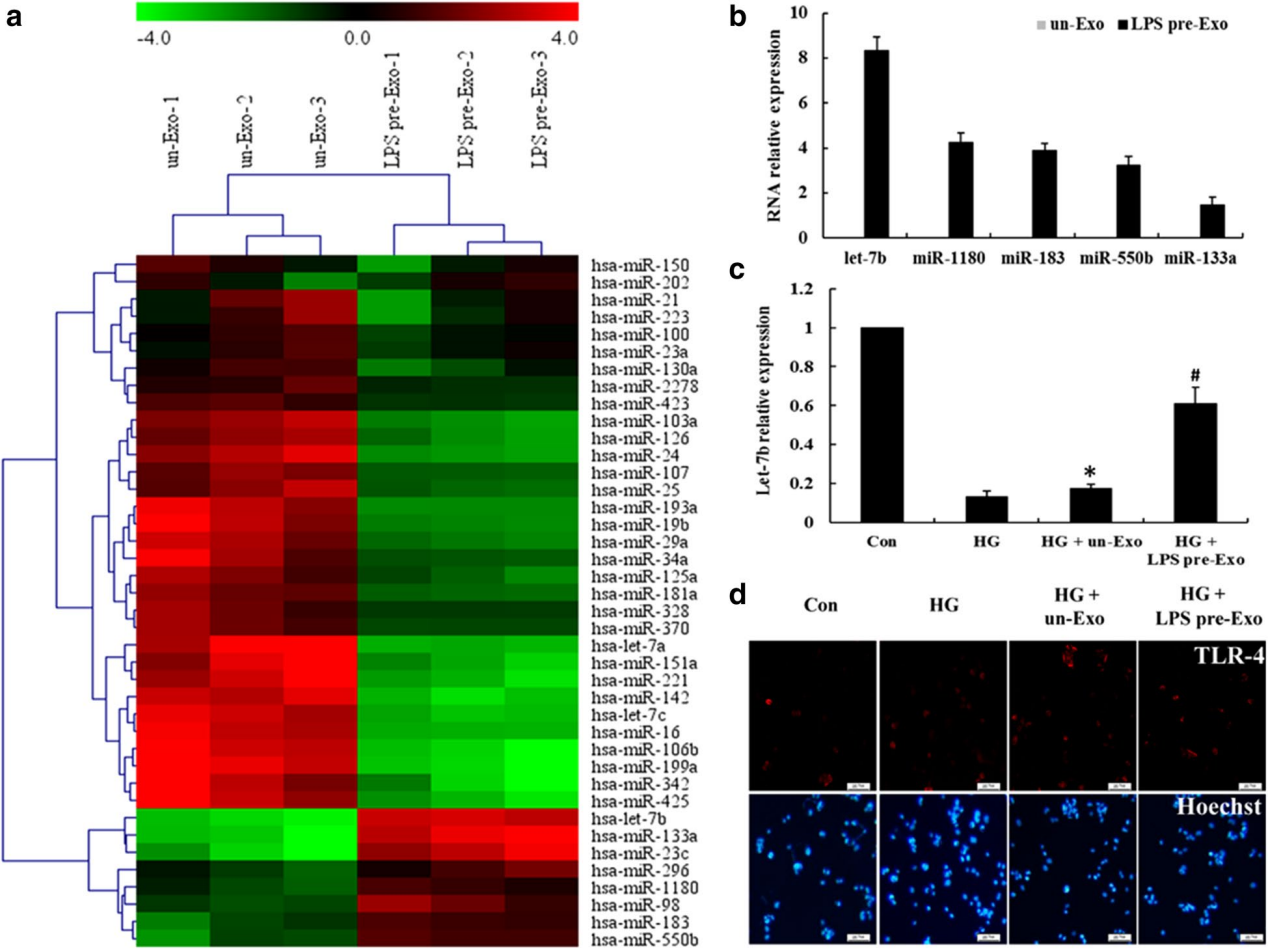
Unsupervised hierarchical cluster analysis of miRNA expression in LPS pre-Exo uncovers let-7b as a regulator of TLR4 in macrophage polarization. **a** miRNA expression profiling on total RNA isolated from un-Exo (n = 3) and LPS pre-Exo (n = 3). A heatmap was generated after supervised hierarchical cluster analysis. Differential miRNA expression is shown by *red* (upregulation) versus *green* (downregulation) intensity (LPS pre-Exo versus un-Exo, twofold change, *p* < 0.05). **b** Validation of unique miRNAs in LPS pre-Exo using real-time RT-PCR. **c** Real-time RT-PCR analysis of let-7b expression in treated THP-1 cells at 48 h. Data are presented as the mean ± SEM of three separate experiments. *Compared with Con, p < 0.05; ^#^compared with HG, p < 0.05. **d** Immunofluorescence staining displayed TLR4 activity in treated THP-1 cells at 48 h

However, LPS pre-Exo have a better effect than un-Exo on the modulation of macrophages plasticity. And exosome-mediated miRNA transport has been proposed to be an essential mechanism that regulates target gene expression for cell-to-cell communication [30], we suggested LPS stimulation may change the composition of MSC exosomes cargo, and some unique miRNA in LPS pre-Exo account for its superior immune-modulatory and regenerative property. So the miRNA expression profiles of LPS pre-Exo were performed using miRNA microarray analysis. Following probe screening and data normalization, we found 40 significantly differentially expressed miRNAs in LPS pre-Exo compared to un-Exo. Differences in miRNA patterns were visualized in a hierarchical clustering plot (heatmap) generated using TIGR multiple experimental viewer software (Fig. 4a). Among them, five miRNAs (let-7b, miR-1180, miR-183, miR-550b, and miR-133a) were only present in LPS pre-Exo. These results were further validated by RT-PCR. Furthermore, we found let-7b has the highest expression level in the five unique miRNAs presented in LPS pre-Exo (Fig. 4b).

Next, to investigate the specific miRNAs and their targets that may be potentially associated with macrophage polarization and inflammation, we used the available databases to search for potential miRNA-targeted genes. Then, we subjected the plausible targets to KEGG pathway enrichment analysis using DAVID tools. According to the Reactome Database, targets of LPS exosomal-specific miRNAs were statistically enriched in genes and proteins participating in cell differentiation, immune regulation, cell proliferation and inflammation (Additional file 1: Table S2). Notably, the let7b/TLR4 pathway has been shown to be the most critical cellular pathway related to the modulation of the immune system and inflammation, and it has been shown to play an important role in both monocyte activation and differentiation after tissue injury [31, 32].

According to the above analysis results, we considered that let-7b maybe play a key role in the M1 to M2 shift in macrophages induced by LPS pre-Exo. Subsequently, we evidenced that THP-1 cells that took up LPS pre-Exo expressed significantly higher levels of let-7b and low levels of TLR4, but no significant changes in these two genes were observed in THP-1 cells taking up un-Exo (Fig. 4c, d). These data indicated that let-7b is abundantly expressed in LPS pre-Exo, and performs its active function through TLR4 in the course of macrophage polarization.

### Let-7b regulates macrophage polarization via TLR4/NF-κB/STAT3/AKT signaling

**Fig. 5:**
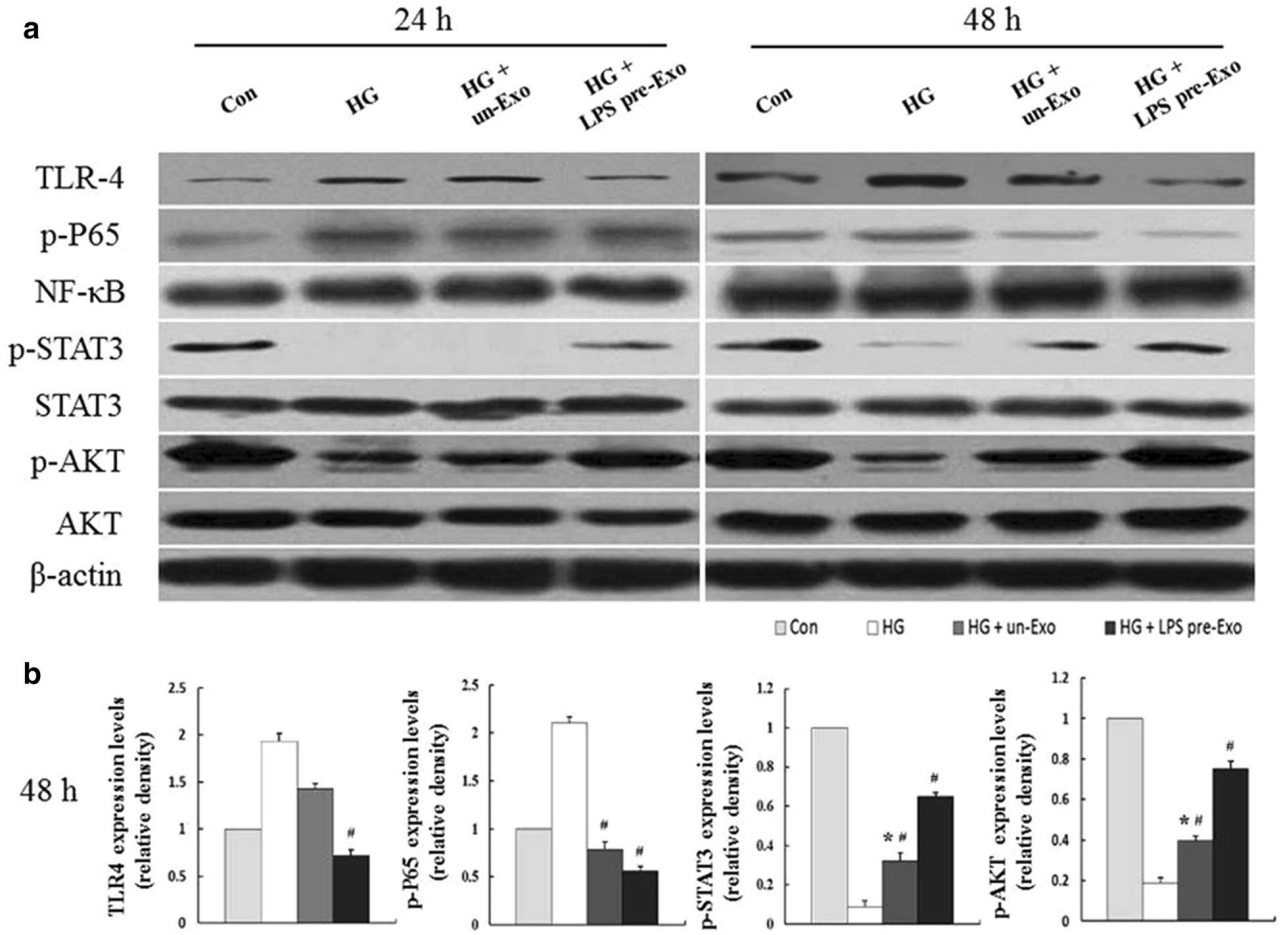
LPS pre-Exo regulated proteins expression in the TLR4/NF-κB/STAT3/AKT signaling pathway. **a** THP-1 cells were pretreated with high gluscose (30 nM) and then cultured with LPS pre-Exo for 48 h. Cell lysates were subjected to Western blot analysis with specific antibody to TLR4, p-P65, NF-κB, p-STAT3, STAT3, p-AKT, AKT, and β-actin. **b** The band intensities were assessed by scanning densitometry. Data are presented as the mean ± SEM of three separate experiments. *Compared with Con, p < 0.05; ^**#**^compared with HG, p < 0.05

**Fig. 6:**
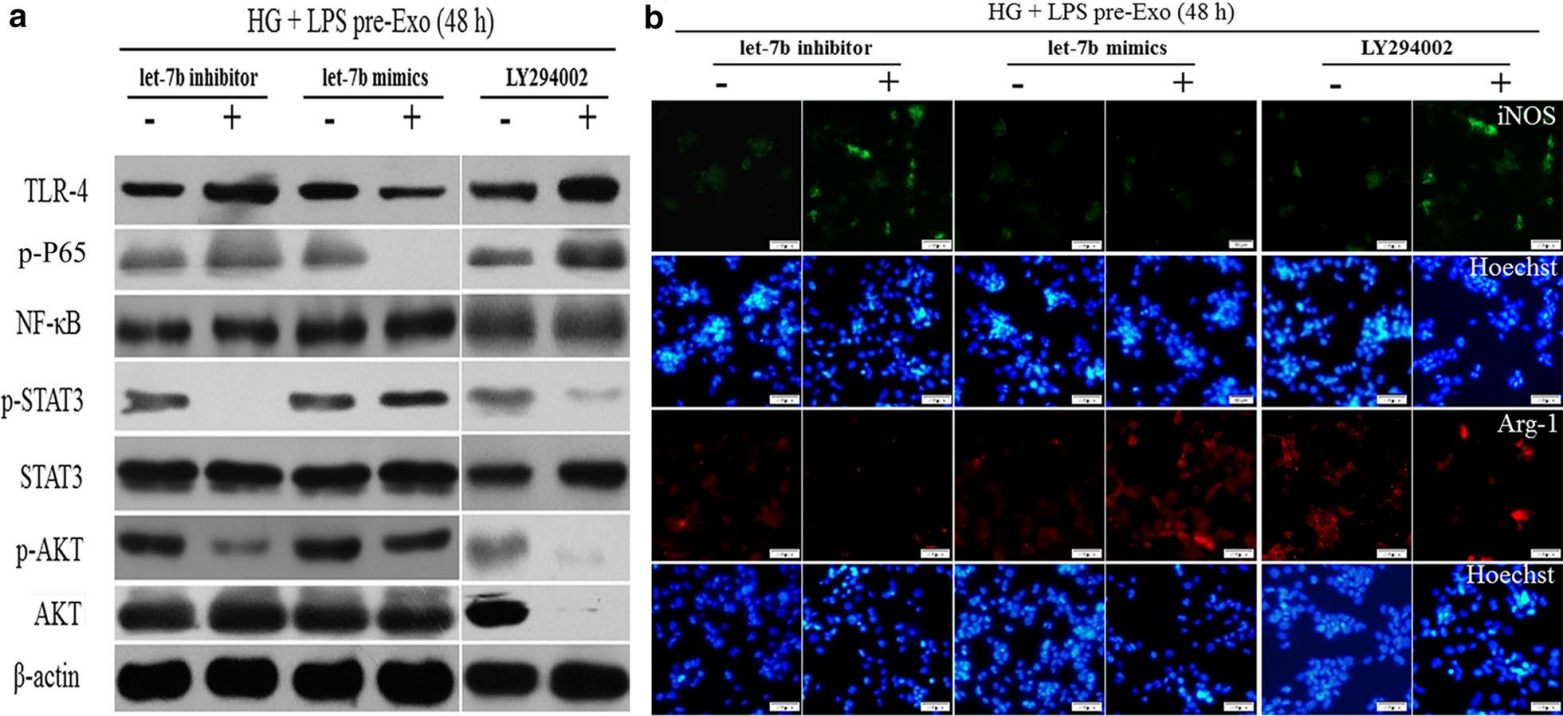
Let-7b is involved in LPS pre-Exo modified macrophage polarization by the TLR4/NF-κB/STAT3/AKT signaling pathway. THP-1 cells were treated with LPS pre-Exo and transfected with the let-7b mimics, let-7b inhibitor or the AKT inhibitor, LY294002. **a** Levels of TLR4, p-P65, NF-κB, p-STAT3, STAT3, p-AKT, AKT protein were detected by western blotting with the respective antibodies. **b** The distribution of macrophage subtype M1 (iNOS *green*) and M2 (Arg1 *red*) were measured by immunofluorescence. *Scale bar* 50 μm

We further analyzed the molecular mechanism whereby let-7b transferred by LPS pre-Exo participates in macrophage plasticity. Western blot analyses revealed that after LPS pre-Exo treatment, the levels of TLR4 and p-P65 expression were significantly decreased. Meanwhile, the expression levels of p-STAT3 and p-AKT were clearly upregulated in a time-dependent manner compared with the control groups at 24 and 48 h (Fig. 5). Furthermore, overexpression of let-7b could augment the regulatory functions of LPS pre-Exo or un-Exo, and inhibition of let-7b in the LPS pre-Exo group had exactly the reverse effect (Fig. 6; Additional file 2: Fig. S1). Remarkably owing to the entire restraint of STAT3 activation in presence of let-7b inhibitor, it is probably let-7b as a key trigger for STAT3 mediated transformation of LPS pre-Exo treated THP-1 cells. We also found that the AKT inhibitor LY294002 prevented the effects of LPS pre-Exo on THP-1 cells (Fig. 6), further indicating that LPS pre-Exo mediate the modulation of macrophage polarization through let-7b via TLR4/NF-κB/STAT3/AKT regulatory signaling.

### LPS pre-Exo relieve inflammation and promote wound healing in diabetic wounds

**Fig. 7:**
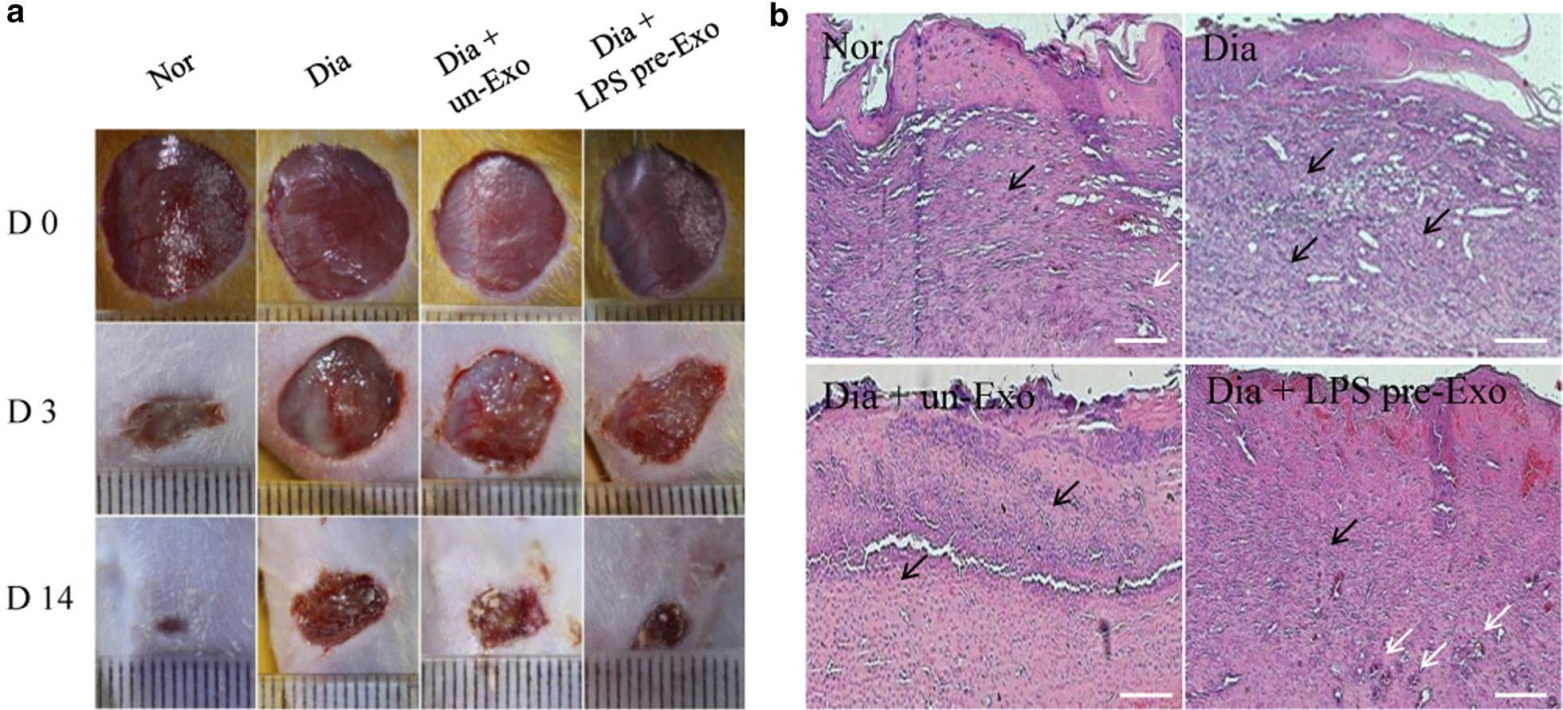
The progression in healing of cutaneous wounds in diabetic rats. **a** Appearances of wounds in different groups at various time points. **b** Histological analyses of cutaneous injury in diabetic rats at 3 days. There are apparently decreased inflammatory cell infiltration (*black arrows*) and promoted the appearance of new small capillaries (*white arrows*) in the Dia + LPS pre-Exo group. *Scale bar* 200 mm

**Fig. 8:**
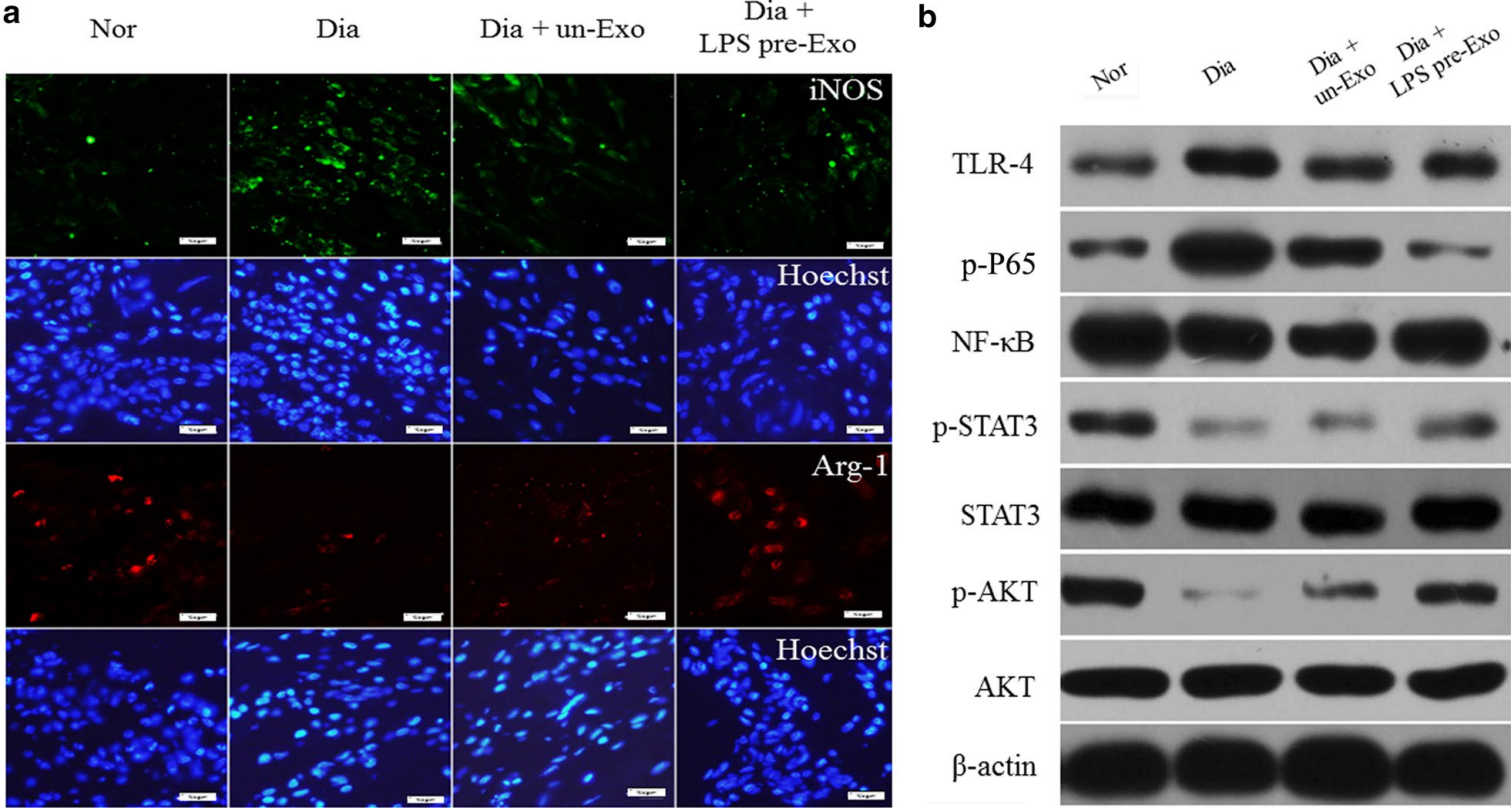
Effects of LPS pre-Exo on wound inflammation in diabetic rats for 3 days. **a** Macrophage phenotype distribution of the wound sites. Immunofluorescence staining for iNOS (M1 *green*), Arg1 (M2 *red*), and nucleus (*blue*). *Scale bar* 50 µm. **b** Western blotting analysis for the TLR4/NF-κB/STAT3/AKT signal pathway

The in vivo curative effects of LPS pre-Exo on inflammation and wound healing were observed and evaluated. We established a cutaneous wound model in streptozotocin-induced diabetic rats, and 60 μg total protein of diverse processing MSCs-generated exosomes (un-Exo or LPS pre-Exo) in 0.5 ml PBS were injected dispersively into the wound edge. The results showed that LPS pre-Exo apparently decreased inflammatory cell infiltration and promoted the appearance of new small capillaries and wound healing (Fig. 7). There appeared to be a high expression level of M2 macrophages and less M1 macrophages in the wound sites at day 3 (Fig. 8a). Western blot analyses revealed low levels of TLR4 and p-P65 expression, but more levels of p-STAT3 and p-AKT, in the wound sites (Fig. 8b). Collectively, these data strongly indicated that LPS pre-Exo can resolve inflammation and enhance wound healing by regulating macrophage polarization.

## Discussion

Recently, LPS preconditioning of MSCs has been an attractive therapeutic approach for the treatment of inflammatory diseases and tissue injury. There is growing evidence that LPS preconditioning obviously enhances the paracrine protective effects and regenerative and repair properties of MSCs [28, 33]. MSCs may release large amounts of exosomes to exchange bio-information between neighboring cells and maintain a dynamic and homeostatic microenvironment for tissue repair [34]. In this paper, we discovered that LPS stimulation increased the production of exosomes from UC-MSCs. Moreover, LPS pre-Exo possessed an apparent advantage for the switch of macrophages to an M2-like profile in inflammatory conditions, and let-7b, distinctively shuttled by LPS pre-Exo, might participate in macrophage polarization by targeting TLR4 to better resolve inflammation and maintain tissue homeostasis. In vivo, LPS pre-Exo greatly alleviated inflammation and enhanced diabetic cutaneous wound healing.

It has become possible to regulate macrophage polarization using interventions that might orchestrate inflammation and optimize wound repair [35, 36]. Macrophages produce a plethora of mediators to balance the inflammatory response in response to environmental stimuli and play an integral role in the successful healing process. Cho et al. demonstrated that MSCs benefit cardiac repair by preferentially polarizing macrophages toward the M2 anti-inflammatory phenotype [37]. Zhu et al. confirmed that human MSC-derived exosomes reduced inflammation and had a similar therapeutic effect on acute lung injury as MSCs [38]. We also found that after treatment of inflammatory THP-1 cells with LPS pre-Exo, they expressed more anti-inflammatory and less pro-inflammatory cytokines. Meanwhile, immunofluorescence also provides evidence that LPS pre-Exo favor broader activation of M2 macrophages in the inflammatory state. Therefore, we suggest that LPS pre-Exo have a superior ability to modulate the balance of M1/M2 macrophages and facilitate wound healing.

Exosomes contain several effective genetic molecules and act as a cell–cell communication vehicle to influence gene expression in recipient cells. Data have shown that the secretion of exosomes is enhanced in cells exposed to a stress environment, and the exosomal contents such as proteins and miRNAs will change distinctively [39]. Given the essential role of the miRNA processing machinery in immune cell development, we revealed that LPS pre-MSCs may secrete exosomes that carry unique “marker signature” of let-7b by microarray analysis and contribute to how the macrophages reach a fully reprogrammed stage. Researches have evidenced that appropriate LPS engagement could reshape the immunomodulation action of MSCs via the TLR4 pathway [40]. Moreover, TLR4 is by now one of the identified target genes of let-7b [41]. Pobezinsky et al. discovered that the let-7 miRNA is pivotal for the terminal differentiation and cytokine effector function of natural killer T cells [42]. So we speculated MSCs expressed moderate TLR4 after LPS stimulation. As a feed-forward loop, LPS pre-MSCs produced let-7b which directly regulates TLR4 expression to maintain a relative homeostasis. Then let-7b was selectively packaged into exosome and transferred to macrophages, negatively regulate TLR4 expression and orchestrate macrophage plasticity, thus fine-tuning the inflammatory response and wound healing.

The let-7 family was the second miRNA family identified as a regulator of developmental timing and cell proliferation; however, it is becoming more apparent that they also mediate immune responses and adjust inflammation [41]. Guo et al. have reported that let-7b, a member of the let-7 family, has differential expression patterns in inflamed tissues compared with healthy controls [43]. In gastric epithelial cells, overexpression of let-7b attenuates NF-κB activity and then regulates downstream genes related to the inflammatory and immune responses by targeting TLR4 [44]. Notably, silencing of the key mediators of TLR4 signaling may provide an important technique for understanding the development of LPS tolerance. There is evidence that LPS priming reprograms TLR4 signaling, whereby NFκB activity is suppressed, anti-inflammatory cytokines are upregulated, and the body is conferred a protective status [45]. Our results are in agreement with these studies, indicating that after LPS preconditioning, MSC-derived exosomes transfer let-7b to THP-1 cells and modulate their behavior through the inhibition of TLR4.

The well-timed transition from a proinflammatory M1 into an alternative M2 state is beneficial for the resolution of chronic inflammation. In this study, high glucose time-dependently induced THP-1 cells to an M1-like state, while LPS pre-Exo switched these M1-like THP-1 cells to anti-inflammatory M2 activation then limited inflammation or boosted tissue repair and regeneration. Several studies have suggested that macrophage polarization is efficiently regulated by various signaling pathways in a strict temporal pattern [46]. A network of various factors, such as multifunctional cytokines, different transcription factors, protein kinases, and receptor signaling, underlies the different forms of macrophage activation. It has been reported that the reciprocal inhibitory crosstalk between NF-κB and STAT3 can modulate the M1/M2 balance and coordinate responses to different microenvironments; NF-κB activation can induce an inflammatory M1 phenotype, and STAT3 activation is important for an anti-inflammatory M2 conversion [47]. The inhibition of NF-κB has also been recently shown to contribute to the process of tolerance and M2 macrophage activation by negatively polarizing M1 macrophages [48]. In addition, STAT3 operates as a pivotal transcriptional repressor that prevents excessive TLR4-driven inflammatory responses. During the anti-inflammatory process, the STAT3 pathway is an important homeostatic mechanism that governs the degree and duration of inflammation, and it can promote the expression of effector genes (IL-10) associated with an M2-like phenotype [49]. Therefore, we examined the activated form of NF-κB and STAT3 then found that LPS pre-Exo treated THP-1 cells have higher expression of p-STAT3, accompanied by lower expression of p-P65. There exists a similar condition in unstimulated THP-1 cells. And the results of western blot are just compatible with the analytical data of macrophage polarization in the LPS pre-Exo treated and unstimulated THP-1 cells. The AKT pathway is another critical or compensatory pathway that can comprise negative feedback loops and establish tolerance in TLR4-mediated immune responses [50]. Androulidaki and colleagues found that AKT can monitor macrophage responsiveness to LPS tolerance by controlling the expression of different miRNAs [51]. In vivo, vasoactive intestinal peptide, an anti-inflammatory neuropeptide, directly reduced TLR4 activities via the AKT pathway and suppressed pro-inflammatory cytokine expression by macrophages [52]. However, to date, there remains minimal data to reveal the role of the AKT pathway in macrophage polarization. Significantly, it has been reported the AKT pathway can suppress TLR4/NF-κB activation and the subsequent inflammatory response [53]. STAT3 can induce AKT activation to influence immune homeostasis and regulate cell differentiation [54]. Thus, we speculate that LPS pre-Exo may involve signaling pathways that directly or indirectly lead to AKT activation via STAT3 and/or to the inhibition of the nuclear translocation of NF-κB, and we found that AKT is indeed highly activated in the LPS pre-Exo-treated group. Furthermore, inhibition of the AKT pathway eliminated the effects of LPS pre-Exo on THP-1 cells. Taken together, we consider that TLR4/NF-κB/STAT3/AKT signaling is an essential pathway for the regulation of macrophage polarization and wound healing via LPS pre-Exo-shuttled let-7b.

In conclusion, we found that LPS pre-Exo have better regulatory properties for macrophage polarization and the resolution of chronic inflammation. Furthermore, the exosome-specific let-7b released from LPS pre-MSCs can concomitantly activate feedback inhibitory mechanisms that restrain the magnitude of inflammatory responses to promote proper wound healing. Identifying the molecular mechanisms suggests that the MSC-mediated transfer of exosomes is a very promising tool for future regenerative and repair therapies.

## Conclusions

In summary, the present study demonstrates MSCs may release plenty of exosomes with superior regulatory and regenerative abilities for the balance of macrophages and the resolution of chronic inflammation after LPS treatment. LPS pre-Exo possessed an apparent advantage for the switch of macrophages to an M2-like profile in inflammatory conditions by shuttling let-7b, and these exosomes carry much immunotherapeutic potential for wound healing.

## Additional files

Additional file 1: Table S1. Primers for quantitative polymerase chain reaction analysis. **Table S2.** Top 15 KEGG pathways targeted by five unique miRNAs in LPS pre-Exo.
Additional file 2: Fig. S1. Let-7b is involved in LPS pre-Exo modified macrophage polarization by the TLR4/NF-κB/STAT3/AKT signaling pathway. THP-1 cells were treated with un-Exo and transfected with the let-7b mimics. Levels of TLR4, p-P65, NF-κB, p-STAT3, STAT3, p-AKT, AKT protein were detected by western blotting with the respective antibodies, and the distribution of macrophage subtype M1 (iNOS, green) and M2 (Arg1, red) were measured by immunofluorescence. Scale bar = 50 μm.
