# Supplementary material for: LPS-preconditioned mesenchymal stromal cells modify macrophage polarization for resolution of chronic inflammation via exosome-shuttled let-7b
Source: J Transl Med. 2015 Sep 19;13:308. doi: 10.1186/s12967-015-0642-6 (PMC4575470; doi:10.1186/s12967-015-0642-6)
Supplement: Supplementary file 1 — Additional file 1: Table S1. Primers for quantitative polymerase chain reaction analysis. Table S2. Top 15 KEGG pathways targeted by five unique miRNAs in LPS pre-Exo. [file 12967_2015_642_MOESM1_ESM.docx]

Table S1. Primers for quantitative polymerase chain reaction analysis

| Gene | 5′-3′ Forward primer | 5′-3′ Reverse primer |
| --- | --- | --- |
| IL-1 | TTCCTGTTGTCTACACCAATGC | CGGGCTTTAAGTGAGTAGGAGA |
| IL-6 | AACAACCTGAACCTTCCAAAGA | TCAAACTCCAAAAGACCAGTGA |
| TNF-α | TGTAGCCCATGTTGTAGCAAAC | TTGAAGAGGACCTGGGAGTAGA |
| IL-10 | GGGAGAACCTGAAGACCCTC | ATAGAGTCGCCACCCTGATG |
| CD163 | CCAACAAGATGCTGGAGTGAC | TGACAGCACTTCCACATTCAAG |
| TGF-β | CCCACAACGAAATCTATGACAA | ACGTGCTGCTCCACTTTTAACT |
| GADPH | AGAACATCATCCCTGCCTCTACT | GATGTCATCATATTTGGCAGGTT |

Table S2. Top 15 KEGG pathways targeted by five unique miRNAs in LPS pre-Exo

| **KEGG pathway** | **Gene count** | ***p*-value** |
| --- | --- | --- |
| T cell receptor signaling pathway | 121 | 2.54e-06 |
| Notch signaling pathway | 108 | 3.1e-02 |
| Cell cycle | 97 | 1.85e-04 |
| TLR4 signaling pathway | 83 | 1.94e-03 |
| Jak-STAT signaling pathway | 75 | 2.76e-02 |
| MAPK signaling pathway | 64 | 4.23e-08 |
| Chemokine signaling pathway | 59 | 1.3e-02 |
| Regulation of actin cytoskeleton | 55 | 2.02e-03 |
| TGF-beta signaling pathway | 53 | 2.54e-06 |
| B cell receptor signaling pathway | 48 | 4.69e-02 |
| Neurotrophin signaling pathway | 45 | 4.86e-02 |
| ECM-receptor interaction | 43 | 5.09e-03 |
| Wnt signaling pathway | 43 | 1.95e-04 |
| Bacterial invasion of epithelial cells | 39 | 1.39e-02 |
| Tight junction | 38 | 2.01e-02 |
