# Supplementary figures and images for: LPS-preconditioned mesenchymal stromal cells modify macrophage polarization for resolution of chronic inflammation via exosome-shuttled let-7b
Source: J Transl Med. 2015 Sep 19;13:308. doi: 10.1186/s12967-015-0642-6 (PMC4575470; doi:10.1186/s12967-015-0642-6)

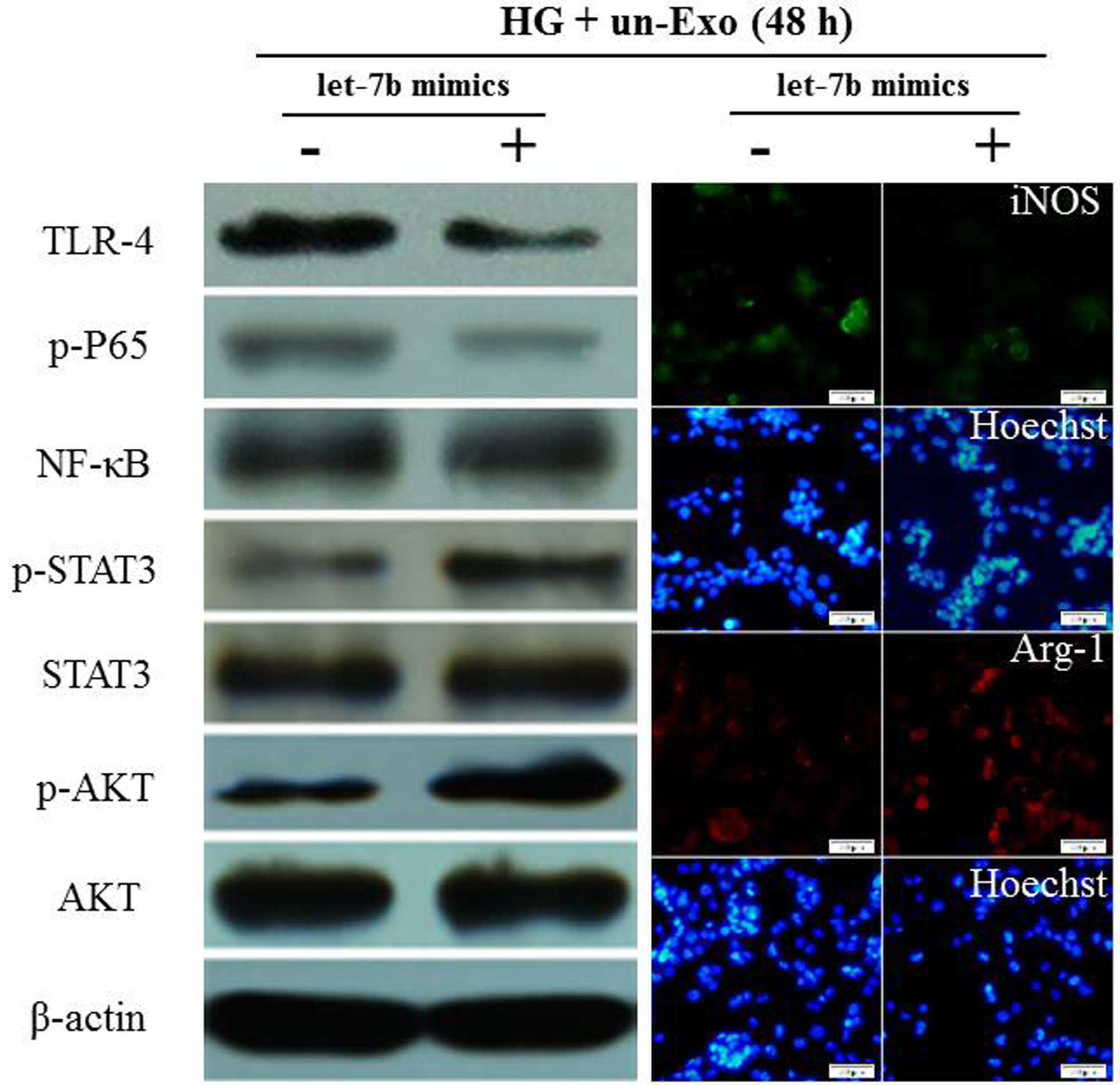

Supplement: Supplementary file 2 — Additional file 2: Fig. S1. Let-7b is involved in LPS pre-Exo modified macrophage polarization by the TLR4/NF-κB/STAT3/AKT signaling pathway. THP-1 cells were treated with un-Exo and transfected with the let-7b mimics. Levels of TLR4, p-P65, NF-κB, p-STAT3, STAT3, p-AKT, AKT protein were detected by western blotting with the respective antibodies, and the distribution of macrophage subtype M1 (iNOS, green) and M2 (Arg1, red) were measured by immunofluorescence. Scale bar = 50 μm. [file 12967_2015_642_MOESM2_ESM.tiff]
